# Overflow Metabolism in *Penicillium ochrochloron* and Causation in Organisms

**DOI:** 10.3389/ffunb.2021.682062

**Published:** 2021-05-12

**Authors:** Wolfgang Burgstaller

**Affiliations:** Retired, Telfs, Austria

**Keywords:** Overflow metabolism, organic acid excretion, causation, *Penicillium ochrochloron*, *Saccharomyces cerevisiae*, *Escherichia coli*

“The organisms parts are reciprocally cause and effect of each others form.” (Kant 1790, Critique of Judgement, § 65).

## Introduction

This article is unorthodox in suggesting a hypothesis which may, at least currently, be inaccessible to experimental tests. But learning from the admired Peter Mitchell, I think that this hypothesis is of heuristic value. My aim is to draw attention to a specific point: That the fungal organism as a whole is the cause of its physiological behavior. I hereby place myself within the tradition of the so-called organismic biology, which was developed, among others, by the Theoretical Biology Club in England in the 1930's (Peterson, 2016). This conclusion imposed itself on me from 30 years of work on the excretion of organic acids by *Penicillium ochrochloron*, i.e., overflow metabolism in the sense of Foster (1949), who first used this term in connection with organic acid excretion by filamentous fungi (the terms overflow metabolism and organic acid excretion, OAE, are used synonymously in this article).

Overflow metabolism, i.e., the excretion of incompletely oxidized metabolites in the presence of oxygen, is a feature of many microorganisms as well as of cancer cells (Warburg effect). Microorganisms in which overflow metabolism was studied in detail are *Klebsiella aerogenes* (Neijssel et al., 2008), *Saccharomyces cerevisiae* (Bruggeman et al., 2020), *Escherichia coli* (Basan et al., 2015; Bruggeman et al., 2020), *Bacillus subtilis* (Dauner et al., 2001), *Streptococcus bovis* (Russell, 2007), *Aspergillus niger* (Karaffa and Kubicek, 2003, 2019; Wierckx et al., 2020), *Aspergillus carbonarius* (Linde et al., 2016) and *Penicillium ochrochloron* (Vrabl et al., 2017).

Especially for *S. cerevisiae* and *E. coli* a detailed experimental and theoretical picture for overflow metabolism exists. No such picture does exist for filamentous fungi despite the importance of fungal overflow metabolism in biotechnology and ecology. I will describe main features of overflow metabolism in *E. coli* and *S. cerevisiae*, argue why this overflow metabolism is fundamentally different from overflow metabolism in *P. ochrochloron* and suggest a hypothesis why *P. ochrochloron* excretes organic acids.

### Overflow Metabolism in *E. coli, S. cerevisiae*, and *K. aerogenes*

The central point concerning *S. cerevisiae* and *E. coli* is that overflow metabolism in glucose limited chemostat cultures starts if the specific growth rate is increased beyond a certain value. Thus, a threshold exists for overflow metabolism. At μ values higher than this threshold experiments showed a linear increase (q, mmol (g dry weight)^−1^ h^−1^) of (i) the excretion of specific metabolites (ethanol, acetate), (ii) the glucose consumption, and (iii) the carbon dioxide production (Bruggeman et al., 2020). Simultaneously the oxygen consumption decreased (Bruggeman et al., 2020). Thus, metabolism changed from respiratory to respirofermentative. These data were won from glucose limited chemostats. This phenomenological description is true regardless of the underlying mechanistic explanation (Shimizu and Matsuoka, 2019).

In *K. aerogenes*, another microorganism well-studied concerning overflow metabolism, the picture for ethanol and acetate excretion seems to be similar to *E. coli* and *S. cerevisiae* (Paca, 1976; Teixeira de Mattos et al., 1982) whereas the scarce results for the dependence of OAE on the dilution rate (Paca, 1976; Neijssel et al., 2008) are closer to the picture found in *P. ochrochloron*.

### Overflow Metabolism in *P. ochrochloron*

Two scenarios will be considered. The description of the two scenarios for *P. ochrochloron* is based on published data, with one exception (Heiss, 2016). Because the focus of experiments changed with the progress of research it has to be accepted that not for all runs all desirable data were available. A similar systematic study of OAE is regrettably neither available for any other filamentous fungus nor for *E. coli* and *S. cerevisiae*. I have deliberately refrained from presenting specific data and only quoted them. To guarantee that the reader can find easily the particular results I am referring to, the quotations include references to specific figures within the quoted articles.

The first scenario is OAE during steady state of chemostat cultures with different nutrient limitations (glucose, ammonium, phosphate). The second scenario is OAE after abrupt changes in environmental conditions such as (i) the addition of inhibitors to ammonium limited chemostat culture (uncouplers, N_2_, SHAM), (ii) the addition of glucose to glucose limited chemostat culture or (iii) the transition from exponential growth to non-exponential biomass increase after the exhaustion of a main nutrient in bioreactor batch culture, or after harvest and resuspension of biomass in an aerated ammonium free glucose solution.

The question that is easiest to answer is how *P. ochrochloron* does excrete organic acids. A more difficult question is, whether or not OAE in *P. ochrochloron* is different from overflow metabolism in *E. coli* and *S. cerevisiae*. The most difficult question is: why *P. ochrochloron* excretes organic acids – this is the question dealing with cause(s) and purpose(s) in organisms. At this point I want to mention that with plant pathogenic and entomopathogenic fungi the why question is answered for, e.g., the fungal excretion of oxalic acid (Palmieri et al., 2019): This excretion is to support infection. The “Why” question in this article refers only to growth of *Penicillium ochrochloron* under artificial laboratory conditions, and not to growth of *P. ochrochloron* or any other fungus in natural habitats.

Main features of OAE by *P. ochrochloron* for the first scenario, i.e., chemostat cultures of *P. ochrochloron* are: (i) in glucose limited chemostats very low excretion of metabolites was observed regardless of the specific growth rate (Gallmetzer and Burgstaller, 2001, Figure 2, Table 1; Gallmetzer and Burgstaller, 2002, Figure 1; Gallmetzer et al., 2002, Figures 1, 3); for example, they showed that even with the very low OAE there is a continuous increase in citrate excretion between a specific growth rate of 0.06 and 0.18 h^−1^;there is thus no threshold value for μ concerning overflow metabolism; (ii) in chemostat culture amounts and pattern of OAE were different with different limiting nutrients (Gallmetzer and Burgstaller, 2001, Figure 2; Gallmetzer and Burgstaller, 2002, Figure 3; Gallmetzer et al., 2002, Figures 1, 2; Vrabl et al., 2017, Figures 3, 4); for example they showed that OAE was strongly increased with ammonium and phosphate limitation compared to glucose limitation; (iii) increased OAE was found together with either an increased or a decreased glucose and oxygen consumption (Gallmetzer and Burgstaller, 2002, Figure 2); (iv) no change from respiratory to respirofermentative metabolism was observed with increasing μ. Thus, the characteristics of overflow metabolism stated for *S. cerevisiae* and *E. coli* do not apply to *P. ochrochloron*.

Second scenario. In NH_4_ limited bioreactor batch cultures OAE increased after the exhaustion of a main nutrient (ammonium, phosphate) combined with a transient decrease in μ, qGlu, qO_2_ and qCO_2_. Biomass formation and qGlu stopped completely for about one hour (Vrabl et al., 2009, Figure 5; Vrabl et al., 2012, Figures 4–6; Heiss, 2016; Vrabl et al., 2019, Figure 4). For example, they showed that the transition from exponential to post exponential growth triggered an increase in OAE.

Pattern and amounts of OAE were also shifted by adding inhibitors like uncouplers and inhibitors of the respiratory chain and the plasma membrane H^+^-ATPase (benzoate, DNP, SHAM, azide, N_2_, sodium orthovanadate; Franz et al., 1993; Burgstaller et al., 1994, Figure 4; Burgstaller et al., 1997, Figure 2; Gallmetzer et al., 1999, Figure 11; Gallmetzer and Burgstaller, 2002, Figure 4; Gallmetzer et al., 2002, Table 1).

Furthermore, increasing extracellular pH (batch und chemostat; Gallmetzer and Burgstaller, 2001, Figure 3; Vrabl et al., 2012, Figures 4, 5) and osmolarity (Gallmetzer and Burgstaller, 2001, Figure 3) increaed OAE.

**Figure 1:**
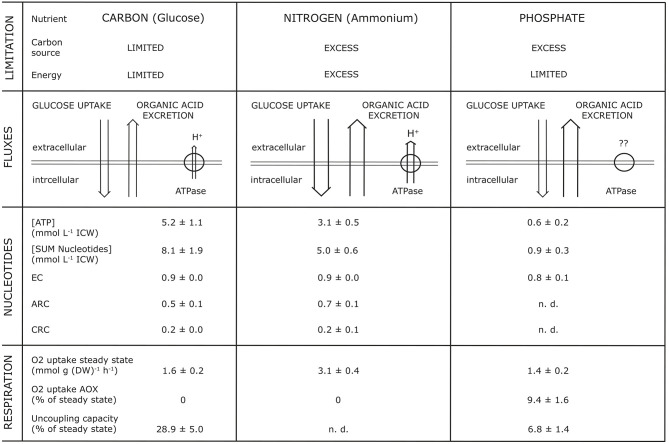
Synopsis of parameters simultaneously analyzed with organic acid excretion at different nutrient limitations (glucose, ammonium, phosphate) during growth of *Penicillium ochrochloron* CBS 123.824 in chemostat culture at a specific growth rate μ of 0.1 h^−1^. EC, Energy Charge; CRC, Catabolic Reduction Charge; ARC, Anabolic Reduction Charge; n.d., not determinable. The Figure is taken from Figure 7 of Vrabl et al. (2017).

Studies into the dynamics of adenine and pyridine nucleotides, and also the energy charge, showed that OAE increased with a decrease of intracellular concentration of nucleotides, whereas the Energy Charge (EC) as well as the Catabolic Reduction Charge (CRC) remained constant (Vrabl et al., 2017, Figure 4). For example, they showed how changes in three essential metabolic levels (the activity of the plasma membrane H^+^-ATPase, the nucleotide concentrations and ratios, the respiratory activity) were related to changes in OAE. These relationships were summarized in a model [Figure 1, taken from Figure 7 in Vrabl et al. (2017)]. It becomes clear that each parameter is individually adjusted to the specific nutritional situation and thus OAE is not determined by one single parameter or reason. It must be assumed, of course, that many more levels of metabolism are involved in OAE.

The resuspension of growing biomass in an aerated ammonium free glucose solution increased OAE (Gallmetzer et al., 1998, Figure 2).

To emphasize it once again: OAE in *P. ochrochloron* differs in my opinion fundamentally from the excretion of ethanol, acetate and lactate by *E. coli, S. cerevisiae* and cancer cells. In consequence OAE in *P. ochrochloron* has nothing to do with a shift from respiratory to fermento-respiratory metabolism (Bruggeman et al., 2020) or with a change in the allocation of resources (Basan et al., 2015; Basan, 2018).

### Hypothesis for the Causation of Overflow Metabolism in *P. ochrochloron*

The big picture that emerges from the total of results gained with *P. ochrochloron* suggests that OAE in *P. ochrochloron* is a more fundamental physiological tool than overflow metabolism of ethanol in *S. cerevisiae* and of acetate in *E. coli*. The hypothesis I suggest is that in *P. ochrochloron* OAE serves as a general means to balance or regulate pool concentrations of metabolites in accordance with extracellular constraints (a specific combination of nutrients; exhaustion of a nutrient; stress factors) and intracellular constraints (activities of metabolic pathways, subcellular compartrmentation). Whether this involves a change in transcriptional patterns is unknown. This balancing of metabolite pool concentrations includes also the reuptake of excreted organic acids in the presence of glucose (Vrabl et al., 2012, Figure 6; Artmann et al., 2020, Figure 4).

The clearest indications for this hypothesis are: (i) both quantity and pattern of OAE depend on the nutrient composition of the growth medium, and thus on the physiological state of the organism as a whole; and (ii) OAE changes when the physiological state changes due to exhaustion of a main nutrient. The pattern of extracellular metabolites can even be used to characterize the physiological state of an organism (Paczia et al., 2012; Granucci et al., 2015; Pinu et al., 2018).

The amounts of enzymes and the substrate concentrations determine metabolic fluxes (Heinemann and Sauer, 2010; Litsios et al., 2018). That pool concentrations of metabolites must be controlled tightly in subcellular compartments of eukaryotic microorganisms is further supported by the observation that TCA cycle metabolites have more regulative functions than thought up to now (Martinez-Reyes and Chandel, 2020). This is true for the mitochondrial matrix and as well for the cytoplasm (Tepper et al., 2013; Wellen and Snyder, 2019; Donati et al., 2021).

For OAE reacting immediately to changes in extracellular and intracellular constraints it must be postulated that transport systems for the efflux of organic acids are constitutively present in the plasma membrane. The activities of these transport proteins are most probable regulated through threshold values and the electrochemical gradient of the respective metabolites. The plasma membrane tranport systems for the efflux of organic acids thus act as some sort of “overflow devices” to keep metabolism functioning. This is supported by results for citrate exporters in several filamentous fungi (Gallmetzer et al., 1998; Yang et al., 2017; Kell, 2019; Odoni et al., 2019; Steiger et al., 2019; Artmann et al., 2020; Kadooka et al., 2020; Nakamura et al., 2021).

From the point of view of this hypothesis it is obvious that in *P. ochrochloron* the “cause” of overflow metabolism is not a single, unidirectional, unilevel reason (Brash, 2020) but is due to the purpose of the whole organism to keep himself functioning in a living state (Tepper et al., 2013; Noble, 2017; Noble et al., 2019; Brash, 2020). Although this seems to be a rather vague statement it can not be formulated in another way. A mathematical formulation was, however, suggested by Noble (2017) and Noble et al. (2019). In line with Brash (2020) metabolic regulations may result from the interactions of many delocalized “control sheets.” These may be for instance, redox control sheets, membrane potential control sheets, law of mass action control sheets, Gibbs energy dissipation control sheets, molecular crowding control sheets and more.

Heuristically this hypothesis means that future research dealing with OAE in filamentous fungi should simultaneously be done on as many metabolic levels as feasible, with special emphasis on subcellular compartmentation. One first attempt approaching this claim can be found in Vrabl et al. (2017).

If we would know: (i) identity and relevant properties (e.g., v_max_ und K_M_) of all efflux and uptake transport systems for organic acids in the plasma membrane; (ii) the activity of the plasma membrane H^+^-ATPase; (iii) the membrane potential across the plasma membrane; (iv) concentration, transported species, and species distribution of all organic acids, in the cytoplasm (not on average!; or still better near the cytoplasmic side of the plasma membrane); (v) complex formations of organic acid anions with magnesium ions in the cytoplasm; and (vi) the biosynthesis rates and consumption rates of organic acids, then we would be able to construct a model from which predictions could be derived and tested. BUT: from the experimental feasibility of this plan we are even further away than from the discovery of life on other planets. So this list only serves to indicate which metabolic levels could be envisaged to begin with future research on OAE in filamentous fungi.

To regulate intracellular metabolite pool concentrations not only via glucose uptake or the activity of catabolic and anabolic pathways, but also by excreting metabolites, increases the robustness of metabolism. One consequence of this view is that part of the extracellular space should be regarded to “belong” to the organism as an “organelle” in just the same way as mitochondria and vacuoles do: “I am I and my circumstances” (Schaechter, 2006).

## Conclusions

The view that the organism as a whole is the cause of ist organismic behavior (including physiological “behavior” like OAE) is shared by an increasing number of scientists (Boogerd et al., 2007; Powell and Dupre, 2009; Pezzulo and Levin, 2016; Noble, 2017; Nicholson and Dupre, 2018; Bizzarri et al., 2019; Noble et al., 2019; Brash, 2020; Levin, 2020; Verhagen et al., 2020). Actually this should be nothing new for a biologist studying organisms (Woodger, 1929; Bertalanffy, 1932; Wieser, 2007; Riedl, 2019). The consequence of this point of view is simple but far-reaching: We should reconsider fundamentally our general view of causation in organisms by including such subjects as multilevel interactions, recursive causation, downward causation, circular causation, and the importance of constraints for causation in organisms (Noble, 2017; Riedl, 2019; Brash, 2020; De Groot et al., 2020). But as we all know such causalities are difficult for us to imagine and understand.

## Author's Note

Dedicated to Paul Erbrich, Wolfgang Wieser, Clifford Slayman and with special gratitude and affection to Reinhold Pöder.

## Author Contributions

The author confirms being the sole contributor of this work and has approved it for publication.

## Conflict of Interest

The author declares that the research was conducted in the absence of any commercial or financial relationships that could be construed as a potential conflict of interest.
